# The Untapped Potential of the Gaming Community: Narrative Review

**DOI:** 10.2196/10161

**Published:** 2018-09-25

**Authors:** William Goodman, Ethna McFerran, Richard Purves, Ian Redpath, Rebecca J Beeken

**Affiliations:** 1 Department of Behavioural Science and Health University College London London United Kingdom; 2 School of Medicine, Dentistry and Biomedical Sciences Queen's University Belfast Belfast United Kingdom; 3 Institute for Social Marketing University of Stirling Stirling United Kingdom; 4 The Behaviouralist London United Kingdom; 5 Leeds Institute of Health Sciences Leeds University Leeds United Kingdom

**Keywords:** video games, advertisements, health behavior

## Abstract

**Background:**

Video gamers are a population at heightened risk of developing obesity due to the sedentary nature of gaming, increased energy intake, and the disruption caused to their sleep. This increases their risk of developing a number of noncommunicable diseases. To date, research seeking to improve health behaviors has focused on developing novel video games to promote behavior change. Although positive results have emerged from this research, large-scale success has been limited due to the lack of transferability to mainstream games and the focus on children and adolescents. The gaming community has a number of unique aspects, which have received comparatively less attention than the development of new video games.

**Objective:**

The purpose of this paper is to highlight under-researched areas that have the potential to encourage positive health behavior among this community.

**Methods:**

A narrative review of the lay and academic literature was conducted to provide context and support to our claims that further research could be beneficial in this area.

**Results:**

Research has found that advertising can have implicit effects on an individual’s memories, which could influence later decisions. However, the effect of the exponential growth of in-game advertisements and the brand sponsorship of gaming events and professional gamers have not been explored in the gaming community. The possibility of using advertising techniques to encourage positive health behaviors within games or at these events has also not been explored. Research suggests that virtual communities can be effective at disseminating health information, but the efficacy of this needs to be explored using known community influencers within the gaming community.

**Conclusions:**

This paper has highlighted a number of potential avenues for the development of interventions within the gaming community. Further research must be conducted alongside game developers to ensure that any in-game developed interventions do not deter gameplay and gamers to ensure that potential approaches are acceptable.

## Introduction

### Background

Obesity has become a major problem, with an estimated 1.9 billion adults worldwide classed as overweight or obese in 2014 [1]. Furthermore, the growing prevalence of childhood obesity is now described by the World Health Organization as a major challenge of the 21st century [2]. Obesity is linked to an increased risk of developing a number of noncommunicable diseases [3] and is the second largest cause of cancer after smoking [4]. Previous research suggests those with a higher body mass index are at an increased risk of up to 17 different cancers [5-7]. An increased intake of energy-dense foods and increasingly sedentary lifestyles are central to the rise in obesity prevalence [1]. The video gamer population is at an increased risk of developing obesity because traditional gaming has now become a sedentary behavior.

Sedentary behavior is also implicated as an independent risk factor for a number of noncommunicable diseases [3]. Epidemiological studies have found that sedentary behavior is a strong risk factor for the development of endometrial [8,9], breast [10,11], and ovarian [12] cancer in women. In men, sedentary behavior is a strong risk factor for the development of colorectal (*borderline* impact for women) [13] and prostate [14] cancer. Sedentary behavior involves performing activities in a sitting or lying position while using low levels of energy, and it is considered separate and distinct from a lack of physical activity [15]. Sedentary behavior includes leisure, work, and transportation activities, and it is commonly measured by quantifying television viewing, video game use, or general screen time [16,17]. Research suggests that sedentary behavior increases the risk of developing obesity [18-20], with those who watch television for more than 20 hours a week more likely to be affected by obesity compared with those who only watch television for 5 hours or less (25% and 14%). Estimates are similar for the use of computers [21].

Video games have also been found to increase energy intake among healthy males. A randomized crossover study found that despite increased levels of energy expenditure while playing video games (mean energy increase compared with the rest state=89 kJ), this was counteracted by higher levels of energy intake after playing video games, resulting in an energy surplus of 335 kJ compared with those who rested for the same period [22]. Furthermore, video games have been implicated in the disruption of sleep when played before bedtime. Research involving healthy, good-sleeping adolescents (N=21) found that there was a highly significant negative correlation between gaming time and sleep duration (*r*=−.92) [23]. Shorter sleep duration has been associated with an increased risk of weight gain among males, and a higher incidence of obesity was observed in males with shorter sleep duration [24]. Those who play a large number of video games may, therefore, represent an *at-risk* population that could benefit from targeted interventions.

The majority of previous research involving gamers has focused solely on the potential for video games to be used as a medium for health behavior change, either by increasing the knowledge and awareness of health behaviors or by including physical activity within the game itself. A large body of research has explored the potential to develop games to improve health [25-27]. A recent review of systematic reviews found that active video games among children could increase levels of physical activity and energy expenditure. Health education games also have the potential to support diabetes-related behavior and dietary change [28]. However, research in this area has focused on children and adolescents, whereas the average gamer age is 35 years in the United States [29]. Furthermore, it is unlikely that long-standing gamers will be motivated to change their gaming habits from mainstream video games to active or educational video games. Therefore, additional opportunities for interventions targeting gamers should be explored.

### Objective

This paper aims to discuss the potential avenues for future research in this area, drawing on the attributes that are unique to the gaming community.

## Methods

This paper comprises a narrative literature review. It draws on lay literature to provide context and descriptive detail and academic literature to support the claims that further research could be beneficial in developing this area for health behavior change. This literature was identified through the following electronic databases: Web of Science, MEDLINE, Google, and Google Scholar. The following keywords were used in various combinations: “video games,” “gamers,” “professional gamers,” “gaming events,” “advertising,” “in-game advertisement,” “vloggers,” “forums,” “online communities,” “virtual communities,” “fast food,” and “marketing.” A *snowball method* was also employed, wherein references from relevant papers were reviewed to identify other relevant papers.

This review will refer to both hardcore and casual gamers as *gamers* due to the lack of a universally accepted definition to objectively categorize these 2 groups [30] and the general lack of distinction between the 2 groups in research to date. The term *gaming community* will be used to cover the areas in which gamers interact, such as video games, gaming events, online forums, and video blogs. Gamers are extremely heterogeneous, representing a range of demographic, social, and behavioral groups, and they span international and cultural divides. Understanding the heterogeneity of gamers and their behaviors and influences and subsequently targeting interventions or marketing would provide a rich avenue of research to pursue; however, this aspect is not explored in this review.

## Results

Several aspects of gaming that could be utilized to engage gamers in health behavior change, including gaming environments, virtual communities, and community influencers, were identified. These features and their ability to be applied to health behavior change are described and discussed below.

### In-Game Advertisement

Companies use marketing to connect consumers with their products and services [31]. There is a range of marketing strategies and tools that companies can employ to promote their brand, with advertising being one of the more expensive approaches. In in-game advertisement (IGA), companies pay to have their brand promoted within a game itself [32,33]. In 2004, companies spent US $34 million on IGA [34]; this spending grew exponentially to US $3.1 billion in 2011 and was expected to reach US $7.2 billion in 2016 [35]. This can occur in different forms such as posters or billboards within the game environment, food that is consumed by a character, or having a character dressed in branded clothing [36]. However, not all games lend themselves to IGA, and it is often only included if it is seen as congruent with the game. With the rise of mobile gaming, advertisements have also started appearing in apps at the top or bottom of the phone screen or as short video clips between levels [37]. These advertisements can be personalized to the user through the use of metadata collected by search engines, social media companies [38], and mobile apps [39], which is thought to make them more effective at reaching their target audience [40,41].

Research has found that IGAs have a strong effect on our implicit memory. For example, 1 study found that participants who played a Formula 1 video game scored significantly higher on a word-fragmentation task of brand names (that were shown in the video game) than those in a control condition [42]. The authors concluded that these effects on implicit memory could in turn influence later decisions. Research has shown this to be the case for television advertising; for example, studies have shown that television advertising for unhealthy food increases positive attitudes toward junk food in children [43] and increases food consumption and time spent eating food among adults [44]. Interestingly, these studies also found that advertising nutritious food showed an increase in positive attitudes toward healthy food and less consumption of food in general.

In 2007, Ofcom, the communications regulator for the United Kingdom, introduced a ban on television advertisements of high fat, salt, or sugar (HFSS) food or drink products for children’s airtime to reduce the exposure of children aged less than 16 years to unhealthy foods [45]. The effectiveness of this ban provided an estimated reduction in children’s exposure to HFSS advertisements by 37% in 2009 compared with 2005 [46]. However, with a migration of viewing habits to Web-based providers, children could still be exposed to HFSS advertising. In 2016, a ruling by the UK advertising regulator extended these restrictions to all nonbroadcast media for children aged less than 16 years or that have an audience made up of 25% of children aged less than 16 years [47]. Although a piece of legislation was expected to come forward in 2017 to address this, video games were still not covered, and therefore, individuals were still exposed to IGA. For example, *Wendy’s* (a burger chain operating primarily in North America) is set to have an IGA for their *Baconactor* (a 950-calorie burger) on billboards, bus shelters, and television screens within the virtual gaming systems of multiple games spanning Xbox, PlayStation 4, and personal computer platforms [48]. Information on the product claims, “The addition of the product was part of a push to add menu items that appeal to the 18- to 34-year-old demographic and expand late-night sales” [49]. Dynamic IGAs, such as those used by Wendy’s, can cost around US $4-12 per 1000 impressions received (1 impression=the advertisement being on screen for 10 seconds). Static IGAs that cannot be changed once the game has been released can cost between US $50,000 and $500,000 [50].

Banning HFSS advertisements in games may be more difficult than with television or the internet, as such a ban cannot be targeted at a certain age bracket and a blanket ban approach may be considered heavy-handed. This ban may also not be possible in certain countries that have laws guaranteeing freedom of speech, such as the United States. However, it may be possible for video games to be utilized in a different way to combat IGA of HFSS. If funds were available, charities (eg, Cancer Research UK or Diabetes UK) could consider counteradvertising and place their own IGA’s, which could target people directly with health-promoting messages or information. Within a collaborative context where charities and game developers share values on an approach, content of such premium games could be developed or adverts may be targeted toward health behavior change to influence implicit memory in gamers. This could be a viable alternative when regulation is not an option. Research has shown that gamers develop negative attitudes to advertisements that are intrusive and incongruent with the game, as it reduces their sense of realism [51,52]. There is, therefore, a need for developers willing to take up the challenge to show how positive advertisements may be effectively included in games to support healthy behaviors and choices.

### Further Promotional Techniques

Brands have diversified their promotional mix beyond advertisements to target the gamers in other unique ways. Viral marketing techniques utilize the internet and already established social networking services and sometimes community influencers to popularize a brand [53]. *Burger King* teamed up with *Sony Spain* to offer the delivery of fast food to gamers who signed up to play with professional gamers online. After completing a game, the professional gamer would then take the order and it would be delivered to the gamer’s door [54]. Viral marketing campaigns have been found to be effective tools for the promotion of a brand [53]. Brands have attempted tie-ins with newly released games by offering discounts and prizes to people that buy their products. For example, with the release of the *Call of Duty: Black Ops III,* gaming prizes including *Double XP* could be won by buying special *Mountain Dew* and *Doritos* products [55]. Research has found that the use of promotions for well-known brands can have a positive impact on its long-term sales prospects [56]. The use of viral marketing and promotions by companies seeking to market their brands cannot be stopped; however, their association with large companies such as *Sony* can be targeted. In recent years, corporate social responsibility has become increasingly important to both the consumer and the company [57]. It can affect the way that consumers evaluate a brand and can act as protection for a company in times of crisis [58,59]. Therefore, the encouragement of collaborative work between health researchers, charities, and corporations would help to serve both sides.

### Gaming Events

Gaming events where gamers and developers gather to play, demonstrate, and discuss games are an extension of the gaming community. They offer additional opportunities for promoting health messages and engagement with audiences. A number of these events attract the video game community, such as comic book conventions, gaming festivals, and eSports tournaments. These typically feature high numbers of sponsors and promotions for energy drinks, fast food, and other high-calorie foods [60].

eSports are video gaming competitions involving mostly professional gamers that contend for prize money. These events are frequently broadcast on television and streamed live over the internet. The final for the League of Legends (a multiplayer online battle arena video game) 2015 World Championship drew a global audience of 36 million viewers [61], which was greater than the 2016 NBA final that drew 31.2 million viewers [62]. eSports events have a range of brands that are marketing partners and sponsor both the events and teams. This is a further promotional technique to increase brand recognition and generate positive publicity within the gaming community. The energy drink Red Bull, in particular, has a strong brand presence in eSports by sponsoring some of the biggest tournaments, teams, and clubs [63]. Monster Energy drink, along with Papa John’s Pizza, sponsors the most successful video gaming team in the world, *Evil Geniuses* [64]. This suggests that these events give advertisers a unique ability to target and engage gamers. Although removing brand investment from these events may be difficult, as with IGA, it may be of significant value to explore promoting advertisements with positive nutrition messages or conducting health and health behavior seminars in the downtime between eSports matches to balance out the effects of other advertisers. In addition, the development of guidelines or regulations for food choice management during these events could encourage organizers to expand the range of food and drink choices on offer. Providing incentives, vouchers, or tokens to eat healthier foods (which could be funded through ticket-pricing adjustments) could also help modify choice, moving to a distinct *nudge* toward healthy eating among this community [65]. Initial work might explore the *appetite* for health marketing at these events and the methods to quantify the potential effects for the video game community.

### Community Influencers

Among many communities, there are those who are considered *influencers*—individuals who have a greater amount of influence over members’ commitment [66]. Professional gamers have emerged as influencers within the gaming community by becoming the best gamers within their respective gaming genres, and they appear to be styled as celebrities of the gaming community [60]. These professional gamers receive investment from companies and in turn wear clothes with the company’s logos for matches, in the same way as active sports such as soccer or tennis. They make videos promoting products and play video games that are live-streamed on the company’s website to attract the attention of the game’s audience by using the gamer as an advertisement [67]. Celebrities have been found to be effective mediums for advertisements of energy-dense and nutrient-poor food products, both for children [68] and adults [69]. This effect is thought to be due to celebrities conferring an implicit benefit and by establishing positive associations with the brand through the transference of qualities of physical appeal and likeability from themselves to the product [70,71]. Again, the absolute removal of this incentive and investment involved for professional gamers might not be achieved easily, as they are often reliant on sponsorship to support their career [72]. Nonetheless, attempting to harness this influence within the community may help to counterbalance unhealthy advertisements. For example, attempting to use the gamers’ influence to create positive attitudes to health behaviors may be a viable option.

Game-related video bloggers (vloggers) have become increasingly popular among gamers. These vloggers post videos of themselves playing games and giving commentary, glitches within games, reviews of games, formal walkthroughs of games, using video games to create films from the content, and *speed runs* of people completing things in the fastest time [73]. An individual known as *PewDiePie* is the most popular vlogger on YouTube with over 54 million subscribers to his channel and was the first YouTube star to reach over 10 billion views; his popularity came from his commentary of video games [73,74]. PewDiePie’s popularity offers significant influence with an engaged audience, with reports of increased game sales when a positive video is posted of the game on his channel [75]. This influence has been found to extend to charity fundraising—raising almost $500,000 for *Water Campaign* by encouraging subscribers to donate [76], demonstrating the potential of these influencers to encourage positive behaviors outside of gaming.

The effect of this *influence* among online communities is borne out in research, which suggests that these influencers provide an important role in the social discourse: encouraging discussion and interaction between members, arranging events, and keeping site content up-to-date [77]. Zhang and Dong [78] have suggested that these influencers can have a lot of sway over the opinions of community members but that this is dependent on their ability to engage the community through sociability and the extent of their knowledge and innovation. Therefore, much as with professional gamers, utilizing these community influencers could help to shape opinions on healthy behavior among gamers.

### Virtual Communities

The aforementioned online social communities have developed in conjunction with many video games [79]. Individuals use these communities to discuss the game and share ideas and goals without the constraints of geographical location [80]. The most frequent gamers, who tend to play multiplayer and online games, can play with other gamers online for an average of 6.5 hours a week and in person for 4.6 hours a week [81], suggesting that the social aspect of gaming plays an important role for those engaged in these communities. Virtual communities have been found to be effective mediums to disseminate health information. They support and encourage discussion among members and can provide places to seek help and emotional support, which can be used to promote behavior change [82,83]. Furthermore, satisfaction with interventions delivered within virtual communities has been found to be significantly higher than that in control conditions [84], although inconsistent results have been found for the effectiveness of moderated virtual communities for behavior change [85]. However, previous research has focused on social networking sites and online patient groups that have typically been set up as interventions for specific studies. Participants in these new communities may therefore not have the same level of trust in the moderator that an already established video game–related community might have. Whether virtual gaming communities are used to discuss health information or if interventions delivered within these communities could promote behavior change needs to be explored.

Research has also begun to investigate the ability of 3-dimensional virtual worlds to act as a more immersive form of online social support [86]. *Second Life* has been studied as one of the most realistic and immersive virtual world’s available [87], where people can create and personalize their avatars that interact with each other and the environment. There is some evidence to suggest that when people practice health behaviors in the virtual world, they are more likely to transfer this to real life [88]. The dissemination of health information and encouragement of positive health behaviors within virtual worlds, like Second Life, could, therefore, prompt behavior change, particularly if accompanied by support from other gamers and/ experts in the game [89]. However, there are *massively multiplayer online role-playing games*, such as World of Warcraft and Star Wars: The Old Republic, which offer less realistic worlds and are instead based on fantasy-oriented worlds. Gamers may interact with these games differently with regard to health matters, if at all. Gaining a greater understanding of how these communities approach both the social aspect of gaming and health issues may be important for the future development of interventions for this population.

## Discussion

The exponential growth of gaming and its supporting online communities is a trend that continues to grow—followed by advertising investment—presenting an opportunity to engage with a key audience. The research outlined in this paper suggests that gamers are already exposed to advertisements that encourage unhealthy behavior both in games and at gaming events. There is, therefore, a need for policy and interventions to attempt to redress these effects and promote healthy behaviors among this group of individuals.

There are points to consider when conducting further research that could have an influence on the development of interventions to influence health behavior change. First, in recent years, the gender gap in those that play video games has narrowed, with women making up over 40% of the gaming population in the United States and Europe [90,91]. However, research suggests that many of the games that are developed are hypersexualized and directed toward heterosexual males [92]. Therefore, any intervention development should consider the gender split to ensure that it has the widest possible application. In addition, the development of interventions should be mindful of the racist undertones that have appeared in some of the most popular video games, such as Grand Theft Auto [93]. The stereotypical approach to character development could exclude certain groups of gamers who may feel judged. This can also extend to individuals who are obese, as research has found that the obesity stigma is not conducive to reducing levels of obesity [94]. Therefore, there is a need to be mindful of any stereotypes when developing interventions.

Moreover, there should be more investigation about the distinction between hardcore and casual gamers and how future interventions could influence each group. Hardcore gamers have been described as those that dedicate a large portion of their leisure time to gaming in comparison with casual gamers [95], and so they may be considered more *at-risk*. However, there is currently no universal definition to objectively categorize the gamer type. A move toward better defining groups of gamers would enable studies to explore whether interventions should target differences in how these groups play games as well as the type of game and other behavioral factors. However, these issues were not explored and were beyond the scope of this review.

Furthermore, although we know that the IGA revenue has risen exponentially in the past decade [35], we do not know how much of this has been spent on the advertisement of HFSS food and drink products. Future research should investigate the occurrence of HFSS advertisements not only within video games but also in the wider areas related to video games, such as gaming events and gaming vloggers. This will give an indication of the diversification of the advertising strategy employed by these companies.

In summary, research to date has focused on the development of games specifically designed to change health, but more could be done to explore opportunities within existing online communities and virtual worlds and utilizing known influencers and methods of advertising to this group. Research in all these areas, conducted in a coordinated way with gamers, vloggers, and game developers, would offer any interventions their best chances of success.

## Abbreviations

IGA: in-game advertisement
HFSS: high fat, salt, or sugar

## References

[1] (2016). World Health Organization.

[2] (2016). World Health Organization.

[3] (2017). World Health Organization.

[4] (2016). Cancer Research UK.

[5] Bhaskaran K, Douglas I, Forbes H, dos-Santos-Silva I, Leon DA, Smeeth L (2014). Body-mass index and risk of 22 specific cancers: a population-based cohort study of 5·24 million UK adults. Lancet.

[6] Lauby-Secretan B, Scoccianti C, Loomis D, Grosse Y, Bianchini F, Straif K, International Agency for Research on Cancer Handbook Working Group (2016). Body fatness and cancer-viewpoint of the IARC working group. N Engl J Med.

[7] Renehan AG, Tyson M, Egger M, Heller RF, Zwahlen M (2008). Body-mass index and incidence of cancer: a systematic review and meta-analysis of prospective observational studies. Lancet.

[8] Gierach GL, Chang S, Brinton LA, Lacey JV, Hollenbeck AR, Schatzkin A, Leitzmann MF (2009). Physical activity, sedentary behavior, and endometrial cancer risk in the NIH-AARP Diet and Health Study. Int J Cancer.

[9] Moore SC, Gierach GL, Schatzkin A, Matthews CE (2010). Physical activity, sedentary behaviours, and the prevention of endometrial cancer. Br J Cancer.

[10] Thune I, Brenn T, Lund E, Gaard M (1997). Physical activity and the risk of breast cancer. N Engl J Med.

[11] Zhou Y, Zhao H, Peng C (2015). Association of sedentary behavior with the risk of breast cancer in women: update meta-analysis of observational studies. Ann Epidemiol.

[12] Patel AV, Rodriguez C, Pavluck AL, Thun MJ, Calle EE (2006). Recreational physical activity and sedentary behavior in relation to ovarian cancer risk in a large cohort of US women. Am J Epidemiol.

[13] Howard RA, Freedman DM, Park Y, Hollenbeck A, Schatzkin A, Leitzmann MF (2008). Physical activity, sedentary behavior, and the risk of colon and rectal cancer in the NIH-AARP Diet and Health Study. Cancer Causes Control.

[14] Orsini N, Bellocco R, Bottai M, Pagano M, Andersson S, Johansson J, Giovannucci E, Wolk A (2009). A prospective study of lifetime physical activity and prostate cancer incidence and mortality. Br J Cancer.

[15] Hamilton M, Hamilton D, Zderic T (2004). Exercise physiology versus inactivity physiology: an essential concept for understanding lipoprotein lipase regulation. Exerc Sport Sci Rev.

[16] (2010). Department of Health, & Department for Children Schools and Families.

[17] Owen N, Healy GN, Matthews CE, Dunstan DW (2010). Too much sitting: the population health science of sedentary behavior. Exerc Sport Sci Rev.

[18] Brown WJ, Miller YD, Miller R (2003). Sitting time and work patterns as indicators of overweight and obesity in Australian adults. Int J Obes Relat Metab Disord.

[19] Epstein LH, Paluch RA, Consalvi A, Riordan K, Scholl T (2002). Effects of manipulating sedentary behavior on physical activity and food intake. J Pediatr.

[20] Robinson TN (1998). Does television cause childhood obesity?. J Am Med Assoc.

[21] Shields M, Tremblay MS (2008). Sedentary behaviour and obesity. Health Rep.

[22] Chaput J, Visby T, Nyby S, Klingenberg L, Gregersen NT, Tremblay A, Astrup A, Sjödin A (2011). Video game playing increases food intake in adolescents: a randomized crossover study. Am J Clin Nutr.

[23] Wolfe J, Kar K, Perry A, Reynolds C, Gradisar M, Short MA (2014). Single night video-game use leads to sleep loss and attention deficits in older adolescents. J Adolesc.

[24] Watanabe M, Kikuchi H, Tanaka K, Takahashi M (2010). Association of short sleep duration with weight gain and obesity at 1-year follow-up: a large-scale prospective study. Sleep.

[25] Cullen KW, Watson K, Baranowski T, Baranowski JH, Zakeri I (2005). Squire's quest: intervention changes occurred at lunch and snack meals. Appetite.

[26] Silk KJ, Sherry J, Winn B, Keesecker N, Horodynski MA, Sayir A (2008). Increasing nutrition literacy: testing the effectiveness of print, web site, and game modalities. J Nutr Educ Behav.

[27] Thompson D, Baranowski T, Buday R, Baranowski J, Thompson V, Jago R, Griffith MJ (2010). Serious video games for health: how behavioral science guided the development of a serious video game. Simul Gaming.

[28] Parisod H, Pakarinen A, Kauhanen L, Aromaa M, Leppänen V, Liukkonen TN, Smed J, Salanterä S (2014). Promoting children's health with digital games: a review of reviews. Games Health J.

[29] (2017). Entertainment Software Association.

[30] Poels Y, Annema J, Verstraete M, Zaman B, De Groof D (2012). Are you a gamer?: A qualitative study on the parameters for categorizing casual and hardcore gamers. IJWI.

[31] Grier SA, Kumanyika S (2010). Targeted marketing and public health. Annu Rev Public Health.

[32] Nelson MR, Yaros RA, Keum H (2006). Examining the influence of telepresence on spectator and player processing of real and fictitious brands in a computer game. J Advert.

[33] Terlutter R, Capella ML (2013). The gamification of advertising: analysis and research directions of in-game advertising, advergames, and advertising in social network games. J Advert.

[34] Burns E (2016). Clickz.

[35] Tassi P (2011). Forbes.

[36] Lorenzon K, Russell CA (2012). From apathy to ambivalence: how is persuasion knowledge reflected in consumers' comments about in-game advertising?. J Market Commun.

[37] (2015). Interactive Advertising Bureau.

[38] Google.

[39] (2015). Share Foundation.

[40] Haghirian P, Madlberger M, Tanuskova A (2005). Increasing advertising value of mobile marketing-an empirical study of antecedents.

[41] Hofacker C, de Ruyter K, Lurie N, Manchanda P, Donaldson J (2016). Gamification and mobile marketing effectiveness. J Inter Market.

[42] Yang M, Roskos-Ewoldsen D, Dinu L, Arpan L (2006). The effectiveness of “in-game” advertising: comparing college students' explicit and implicit memory for brand names. J Advert.

[43] Dixon HG, Scully ML, Wakefield MA, White VM, Crawford DA (2007). The effects of television advertisements for junk food versus nutritious food on children's food attitudes and preferences. Soc Sci Med.

[44] Harris JL, Bargh JA, Brownell KD (2009). Priming effects of television food advertising on eating behavior. Health Psychol.

[45] (2007). Ofcom.

[46] (2010). Ofcom.

[47] (2016). Committees of Advertising Practice.

[48] (2017). Ricki.

[49] (2017). Baconator.

[50] Howard J Rapidfire.

[51] Hernandez M, Chapa S (2010). Adolescents, advergames and snack foods: effects of positive affect and experience on memory and choice. J Market Commun.

[52] Lewis B, Porter L (2010). In-game advertising effects: examining player perceptions of advertising schema congruity in a massively multiplayer online role-playing game. JIAD.

[53] Toubia O, Stephen A, Freud A (2011). Viral marketing: a large-scale field experiment. Economics, Management & Financial Markets.

[54] Diaz A (2017). Ad Age.

[55] (2015). Pepsico.

[56] Slotegraaf R, Pauwels K (2008). The impact of brand equity and innovation on the long-term effectiveness of promotions. J Mark Res.

[57] Tennant F (2015). Financier Worldwide Magazine.

[58] Lins KV, Servaes H, Tamayo A (2015). Social capital, trust, and firm performance: the value of corporate social responsibility during the financial crisis. J Finance.

[59] Klein J, Dawar N (2004). Corporate social responsibility and consumers' attributions and brand evaluations in a product-harm crisis. IJRM.

[60] Jin DY, Chee F (2008). Age of new media empires: a critical interpretation of the Korean online game industry. Games and Culture.

[61] (2015). LoL Esports.

[62] (2016). Nielsen.

[63] Kresse C (2016). Esports Marketing Blog.

[64] Monster Energy.

[65] Arno A, Thomas S (2016). The efficacy of nudge theory strategies in influencing adult dietary behaviour: a systematic review and meta-analysis. BMC Public Health.

[66] Koh J, Kim YG (2004). Sense of virtual community: a conceptual framework and empirical validation. Int J Electron Comm.

[67] Paksoy H, Ozcalici M, Yalcin Y (2015). Walking advertisements: a research on athletes in Turkey. IRMBR.

[68] Dixon H, Scully M, Niven P, Kelly B, Chapman K, Donovan R, Martin J, Baur LA, Crawford D, Wakefield M (2014). Effects of nutrient content claims, sports celebrity endorsements and premium offers on pre-adolescent children's food preferences: experimental research. Pediatr Obes.

[69] Dixon H, Scully M, Wakefield M, Kelly B, Chapman K, Donovan R (2011). Parent's responses to nutrient claims and sports celebrity endorsements on energy-dense and nutrient-poor foods: an experimental study. Public Health Nutr.

[70] Kamins M (1990). An investigation into the “match-up” hypothesis in celebrity advertising: when beauty may be only skin deep. J Advert.

[71] Ohanian R (1970). The impact of celebrity spokespersons' perceived image on consumers' intention to purchase. J Advert Res.

[72] Beck K (2016). Mashable.

[73] Weber E (2014). Zefr.

[74] McAlone N (2017). Business Insider.

[75] Dewey C (2015). Washington Post.

[76] Kjellberg F (2013). Charity: Water.

[77] Rothaermel F, Sugiyama S (2016). Virtual internet communities and commercial success: individual and community-level theory grounded in the atypical case of TimeZone.com. JOM.

[78] Zhang X, Dong D (2008). Ways of identifying the opinion leaders in virtual communities. IJBM.

[79] Weibel D, Wissmath B, Habegger S, Steiner Y, Groner R (2008). Playing online games against computer- vs. human-controlled opponents: effects on presence, flow, and enjoyment. Comput Hum Behav.

[80] Kardaras D, Karakostas B, Papathanassiou E (2003). The potential of virtual communities in the insurance industry in the UK and Greece. IJIM.

[81] Entertainment Software Association (2016). Essential facts about the computer and video game industry.

[82] de la Peña A, Quintanilla C (2015). Share, like and achieve: the power of Facebook to reach health-related goals. IJCS.

[83] Ginossar T (2008). Online participation: a content analysis of differences in utilization of two online cancer communities by men and women, patients and family members. Health Commun.

[84] Salzer MS, Palmer SC, Kaplan K, Brusilovskiy E, Ten HT, Hampshire M, Metz J, Coyne JC (2010). A randomized, controlled study of internet peer-to-peer interactions among women newly diagnosed with breast cancer. Psychooncology.

[85] Eysenbach G, Powell J, Englesakis M, Rizo C, Stern A (2004). Health related virtual communities and electronic support groups: systematic review of the effects of online peer to peer interactions. BMJ.

[86] Davis DZ, Calitz W (2016). Finding Healthcare Support in Online Communities: An Exploration of the Evolution and Efficacy of Virtual Support Groups. Handbook on 3D3C Platforms.

[87] Partala T (2011). Psychological needs and virtual worlds: case second life. Int J Hum Comput Stud.

[88] (2008). AIS Health.

[89] Beard L, Wilson K, Morra D, Keelan J (2009). A survey of health-related activities on second life. J Med Internet Res.

[90] (2015). Entertainment Software Association.

[91] (2012). Interactive Software Federation of Europe.

[92] Burgess M, Stermer S, Burgess S (2007). Sex, lies, and video games: the portrayal of male and female characters on video game covers. Sex Roles.

[93] Leonard D (2003). “Live in your world, play in ours”: race, video games, and consuming the other. SIMILE.

[94] Puhl RM, Heuer CA (2010). Obesity stigma: important considerations for public health. Am J Public Health.

[95] Bosser A, Nakatsu R (2006). Hardcore gamers and casual gamers playing online together.

